# Digital twin in poultry production: A framework for enhancing workforce to job market readiness

**DOI:** 10.1016/j.psj.2025.105497

**Published:** 2025-06-28

**Authors:** Farid S. Nassar, Ahmed O. Abbas

**Affiliations:** Department of Animal and Fish Production, College of Agricultural and Food Sciences, King Faisal University, Al-Ahsa, Saudi Arabia

**Keywords:** Digital twin, Poultry farm management, Virtual simulation, Agriculture technologies, Job Market

## Abstract

The integration of advanced technologies in agricultural education is essential to bridging the gap between theoretical knowledge and practical application. Digital twin (DT) technology stands out as a promising tool for simulating complex environments such as poultry farms, offering students immersive opportunities to engage with farm management processes without the need for physical presence in live production settings. This study explores the potential of DT-based virtual simulations to enhance student learning experiences and engagement in poultry farm management within higher education programs, while also examining their role in preparing graduates for the job market and influencing student enrollment in poultry science disciplines. A qualitative descriptive approach was adopted, including a review of relevant literature, to evaluate the impact of DT technology on various aspects of animal production, poultry science, and educational practices in higher education institutions. The findings reveal that incorporating DT into poultry-related academic programs creates interactive, safe environments where students can participate in activities such as ventilation control, feeding, disease monitoring, and performance analysis. These simulations contribute to deeper cognitive understanding, improved decision-making skills, and heightened motivation among current students, while equipping graduates with high-level competencies suited to a competitive labor market. Furthermore, the use of DT technology is expected to have a positive impact on students’ perceptions, making poultry science programs more attractive and accessible. Based on these insights, the study recommends the systematic integration of DT technology into poultry science curricula and programs, given its powerful role in enhancing interactive learning, stimulating student interest, bridging the gap between theory and practice, improving graduate readiness for the job market, and increasing student enrollment through an engaging and realistic educational experience.

## Introduction

Global population growth is accelerating, with projections indicating an increase of approximately 2 billion individuals within the next 27 years, reaching an estimated 9.7 billion by the year 2050 (United Nations, 2019). In addition, Bist et al. (2024) noted that the rapid population growth has intensified pressure on global food systems to meet the growing demand. Among the various sectors contributing to food security, the poultry industry stands out as a primary supplier, having contributed close to 40 % of total meat production worldwide in 2020 and consistently over the past 30 years. Furthermore, egg production has experienced a substantial growth of 150 %, largely driven by developments in Asia. Notably, China accounts for 38 % of global egg production, while the United States contributes 17 % of the poultry output and remains the leading global producer of poultry meat. The industry's expansion is influenced by demographic changes, rising incomes, and increased urbanization, positioning poultry production as a cornerstone in satisfying global protein requirements nevertheless, despite its rapid growth, the sector faces pressing issues related to sustainability, efficient resource utilization, and environmental responsibility (Bist et al., 2024).

At present, with the significant expansion of livestock and poultry farming, digital management has become an increasingly vital necessity to ensure production efficiency. It plays a fundamental role in poultry production by enhancing performance and productivity. With advancements in information technology and artificial intelligence, Digital Twin (DT) systems are gradually being adopted in poultry farm management, enabling more accurate and effective monitoring and optimization of operations (Jia et al., 2025).

The rapid advancement of technology has reshaped everyday life, particularly with the emergence of artificial intelligence, which has influenced various sectors, including industry, agriculture, medicine, and education. In this context, DT has gained significant traction, emerging as a transformative concept in recent years. Technological advancements in the internet of things (IoT), artificial intelligence (AI), data analytics, and cloud computing have facilitated the collection, analysis, and processing of vast amounts of data more efficiently and cost-effectively. Consequently, DT is now being deployed at scale, offering valuable insights and data-driven decision support. These virtual models enable organizations to simulate scenarios, test various strategies, and assess the impact of potential changes before implementing them in the real world, thereby minimizing risks and uncertainties associated with decision-making processes. The applications of DT extend beyond specific industries, encompassing diverse domains such as manufacturing, healthcare, transportation, smart cities, and energy management. This broad applicability has fueled growing interest in the technology. The academic sector has also recognized the potential of DT, with ongoing efforts to integrate them into various aspects of education, including teaching, research, and administrative processes. As technology continues to evolve, novel applications will likely emerge, transforming how higher education institutions (HEIs) approach instruction, learning, research, and institutional management (Chande, 2024).

In the second decade of the 21st century, digital transformation has become a strategic priority for HEIs. It is a vital step toward achieving leadership and maintaining competitiveness. This transformation affects various institutional dimensions, including teaching, administration, infrastructure, research, and community engagement. More than a mere technological upgrade, it represents a fundamental shift in institutional culture, administrative structures, and pedagogical strategies. Successful implementation requires comprehensive restructuring and an integrated approach centered on individuals and their role in institutional and societal advancement (Benavides et al., 2020).

Although digital transformation has significantly influenced HEIs across various domains, such as teaching, administration, and digital infrastructure, existing research still lacks in-depth exploration of how DT technology can be strategically employed as an educational tool within agricultural education, particularly in poultry farm management. This technology offers virtual environments that accurately simulate real-world conditions, enabling students to interact with realistic scenarios involving ventilation, feeding, health monitoring, and performance analysis, without requiring physical presence in live production settings. Such an approach enhances practical understanding, supports the development of decision-making skills, and boosts learning motivation, thereby equipping students more effectively for the labor market. Consequently, DT technology represents a vital foundation for modernizing educational programs in HEIs, supporting the sustainability of the poultry sector by preparing highly qualified graduates with the technical and cognitive skills necessary to meet the evolving challenges of the industry.

To address these challenges, it is essential to cultivate a workforce that possesses both deep knowledge and practical skills tailored to the dynamic demands of the poultry industry. Moreover, HEIs are therefore expected to play a strategic role by fostering educational environments that merge academic instruction with hands-on application. Embracing innovations such as DT technologies can provide students with immersive, interactive simulations of poultry farm operations. These tools offer a safe and realistic context for exploring management scenarios, optimizing decisions, and understanding complex processes, without requiring physical access to operational farms. By doing so, educational institutions can better prepare graduates for real-world challenges, ultimately enhancing both individual employability and the sustainability of poultry production systems. Accordingly, this study seeks to address the following key questions:

Q1- What is the meaning of DT and what are its characteristics?
Q2-How Can DT technology be employed to simulate poultry production processes in a way that serves the goals of higher education?
Q3- What is the impact of implementing DT-based educational framework on enhancing student engagement, improving the learning experience, and increasing job market readiness?
Q4- What are the key challenges and opportunities in integrating DT technology into poultry education and livestock production systems?

In light of the rapid digital transformation and the increasing competition in the higher education sector, it has become essential to adopt integrated strategies that enhance student experiences and develop their practical skills in poultry farm management and the various branches of poultry science. This study highlights the importance of integrating DT technology as a strategic approach to enriching student learning by allowing them to engage with realistic simulations of farm production processes or practical laboratory components. This interaction enables students to gain a deeper understanding of production systems, related disciplines, and the challenges they may face in the job market as graduates. Additionally, it can play a pivotal role in attracting pre-university students to pursue academic programs in poultry science by correcting misconceptions and shaping a positive perception of the career paths and fields associated with poultry sciences. By leveraging DT technology, higher education institutions can offer innovative learning environments that support the development of the technical and cognitive skills required in the poultry industry. Consequently, further applied research is needed to explore how this technology can be effectively integrated into educational curricula and its actual impact on student learning and employability in the poultry sector.

## Material and methods

This study employed a qualitative descriptive research approach to explore Digital Twin (DT) in poultry production as a higher education framework to enhance student learning experience and professional skills for the job market. According to Neuman (2014), descriptive research is characterized by its ability to "depict the specific details of a situation, social setting, or relationship" and "commences with a clearly defined issue or question, aiming to describe it with precision," focusing primarily on 'how' and 'who' inquiries. Lambert & Lambert (2012) emphasize that qualitative research methodologies, including phenomenology and grounded theory, serve both descriptive and explanatory functions. They advocate for the use of the term "qualitative descriptive research" to accurately define studies that do not strictly align with other qualitative approaches, such as ethnography or phenomenology. Qualitative descriptive research is rooted in naturalistic inquiry, which seeks to examine phenomena as they exist within their natural context (Lambert and Lambert, 2012). Given the nature of this study, which aims to identify key characteristics associated with DT in poultry production as a higher education framework to enhance student learning experience and professional skills for the job market, it aligns with the qualitative descriptive research paradigm. Furthermore, to achieve the study’s objectives, a comprehensive content analysis of digital and printed resources pertinent to the subject matter was conducted (Bowen, 2009). Bowen (2009) outlines three key phases of content analysis: skimming, thorough reading, and interpretation. Content analysis allows for the systematic breakdown of reading and writing into smaller, manageable components, facilitating the identification of key themes and patterns within the materials (Weber, 1990). The primary objective of this analytical approach is to extract fundamental insights by recognizing recurring themes and trends in the available literature and data (Patton, 2002). Moreover, the methodology adopted in the present study comprised four key phases: The first phase involved conducting a systematic search across reputable academic databases, including Scopus, Web of Science, and Google Scholar, using key search terms such as “Digital Twin,” “academic programs,” “higher education,” “skills development,” and “labor market.” The search focused on peer-reviewed articles, institutional reports, and empirical studies published in English over the past five years, supplemented by foundational references to provide a solid theoretical framework. The second phase centered on establishing precise inclusion and exclusion criteria. Studies were included if they directly addressed the use of DT technology to enhance educational and professional outcomes for students in higher education, facilitate the development of practical, job-ready skills, and support productivity improvements in factories and livestock production farms. Studies were excluded if they were not directly related to higher education or production settings, or if they lacked clear methodologies or empirical evidence. In the third phase, information were extracted and analyzed from the selected sources, capturing the main focus of each study, key findings, practical applications of DT technology in higher education, as well as identified challenges and recommendations. This information was synthesized to uncover the core benefits and determine the role of technology in advancing education and professional skill development within the poultry sector. Finally, the fourth phase involved an in-depth analysis of the extracted findings to understand how DT technology can be employed as an effective educational framework to enhance student learning experiences and graduate competencies in higher education and the poultry industry. The findings were discussed, and future opportunities for advancing higher education and poultry production were explored. This structured approach was designed to deliver a comprehensive and rigorous assessment of the role of DT technology in supporting higher education and meeting the evolving demands of the poultry sector labor market.

## Results and discussion

### Understanding DT: meaning and key characteristics

The global population is projected to reach 8.5 billion by 2030, 9.7 billion by 2050, and 10.4 billion by 2100 (United Nation, 2022). This significant growth is expected to lead to a sharp increase in food demand. To meet this growing demand, global agricultural production will need to at least double by 2050 (Tilman et al., 2011; Alexandratos and Bruinsma, 2012).

In response to these challenges, a variety of agricultural technologies integrated with Information and Communication Technology are being developed to ensure sufficient food production for the expanding population (Farooq et al., 2022). Among the most important of these innovations is Agriculture 4.0, which plays a pivotal role in advancing both industrial and agricultural sectors. Agriculture 4.0 leverages cutting-edge technologies such as edge computing (Zhang et al., 2022), AI (Smith, 2018), real-time sensing, wireless sensor networks (Loukatos and Arvanitis, 2021), and IoT (Paraforos and Griepentrog, 2021), all of which enable data-driven agricultural practices. Moreover, building on this technological foundation, Agriculture 5.0 (Kaklauskas, 2018) shifts the focus toward sustainability, fostering growing interest in DT and their applications in the agricultural sector (Tzachor et al., 2022).

On the other hand, DT is a virtual representation of a physical entity, such as an animal, system, or process, that is activated through real-time data exchange. It mirrors the status and behavior of its physical counterpart, allowing for simulation, analysis, and informed decision-making. DT rely on advanced technologies such as IoT, AI, and cloud computing (Arulmozhi et al., 2024). While the concept of DT is not new, it has gained significant attention over the past two decades. The first practical application of DT technology was made by NASA during the Apollo space program. Two identical spacecraft were created: one launched into space, and the other remained on Earth to simulate and replicate the conditions the spacecraft faced during its flight (Tarek et al., 2025).

The concept of DT was documented in a white paper published in 2014 (Grieves, 2014). Since then, various definitions have emerged. According to (Chen, 2017), DT is a computerized representation of a system or device that encapsulates all its functional characteristics and remains continuously linked to its operational components. Unlike a simple virtual simulation, the DT offers a comprehensive understanding of outcomes and provides deep insights into the physical system. Moreover, Liu et al. (2018) defined the DT as a living, dynamic model of a physical asset that is continuously updated based on real-time data collected from the asset itself. Moreover, DT can generally be defined as the creation of a virtual model representing a physical asset. This model can encompass various entities such as vehicles, buildings, individuals, cities, companies, and systems, and operates in real or near-real time. The virtual model may focus on specific aspects of the asset, such as financial records of a company, or represent all dimensions, including geographic planning, human resources, financial data, relationships with other entities, and physical components like storage facilities, machinery, and tools.

Developing a comprehensive DT that captures all aspects of an asset is a complex, expensive, and labor-intensive process. This highlights the need for a general framework for DT that can be easily applied across various fields. The use of DT technology has grown significantly in recent years, driven by enabling technologies such as big data, IoT, AI and machine learning, and cloud computing. These technologies play a crucial role in ensuring accurate collection, transmission, storage, and analysis of data, especially given the vast amounts of data generated by IoT sensors (Fuller et al., 2020; Rathore et al., 2021) . As a result, DT technologies are now being applied across a wide array of fields including smart manufacturing, medical, healthcare, transportation, education, business, and smart cities (Tarek et al., 2025).

Communication within cyber-physical systems occurs through seamless interaction among machines, devices, and humans, integrating both physical and digital elements. DT act as virtual counterparts to physical products, simulating their real-world behavior in real time, which enables the identification of faults and potential inefficiencies. According to M. Greaves, the DT model consists of three primary components: physical products in the real world, virtual products in the digital space, and the flow of data and information connecting the two (Grieves et al., 2014). These virtual representations facilitate comprehensive analysis, testing of new functions, and simulation of scenarios that would be difficult or costly to replicate in the physical world (Bréchet et al., 2019).

The primary goals of the DT are to reduce operational costs, enhance maintenance efficiency, drive innovation in products and services, and optimize processes by improving components or reducing delays in interconnected subsystems (Selim et al., 2024). This, the use of DT contributes to improving productivity and agricultural efficiency by simulating processes in real-time and continuously analyzing data, which aids in making informed decisions. It also enhances environmental sustainability by reducing waste and the excessive use of resources, while promoting innovation and continuous development in the agricultural sector. Additionally, DT help in responding quickly to crises such as climate changes or disease control, improving planning and management while reducing costs and increasing economic returns.

### Employing DT technology to simulate poultry production processes: an innovative educational tool for advancing higher education goals

The shift from traditional agricultural practices to advanced techniques, such as smart farming, is crucial for enhancing productivity. Consequently, livestock production systems are increasingly adopting smart farming approaches, driven by innovations in cloud computing, IoT, big data, machine learning, augmented reality, and robotics. A key component of this advanced digital agriculture is DT, which serves as a virtual replica of a physical entity, linked to it via real-time data exchange. The DT consistently mirrors the condition of its physical counterpart, facilitating synchronized monitoring and interaction between the digital and physical systems (Arulmozhi et al., 2024).

The digital revolution has brought about significant innovations and increased efficiency across various sectors. One of the most impactful advancements in agriculture is the DT, which offers an advanced method for monitoring and understanding agricultural systems. In addition, DT is a virtual representation of physical assets or processes, allowing for real-time monitoring, predictive analytics, and improved decision-making (Pylianidis et al., 2021; Menon et al., 2023). These systems provide valuable insights and unlock numerous opportunities for enhancement. In the livestock sector, where animal health, welfare, and productivity are of utmost importance, DT technology holds the potential to revolutionize traditional farming practices and address contemporary challenges (de Koning et al., 2023).

The physical agricultural system, where actual agricultural operations take place, is a dynamic and complex environment. It includes crucial information and characteristics of various objects or devices, such as shape, location, cooling systems, material properties, and biological components (Mallinger et al., 2022; Verdouw et al., 2021). This system may represent an individual component or the entire structure, along with its subsystems, within the real-world context. It encompasses living organisms like animals, farm infrastructure, and resources such as buildings (Jeong et al., 2023), feeding mechanisms (Raba et al., 2021), and animal populations (Agnusdei et al., 2021; Symeonaki et al., 2024; Neethirajan et al., 2021). Accurate data from these physical systems is collected through measurement technologies and sensors, which are vital for the functionality of DT. Without a physical counterpart, DT is just a conceptual model. Therefore, the boundaries of any DT are determined by the physical system it represents (Arulmozhi et al., 2024).

In the realm of smart livestock management, DT technology offers a broad array of applications, including animal tracking, disease detection through sensors, behavioral monitoring, environmental control via building sensors (for temperature, humidity, and ammonia), energy management in animal housing, and optimization of the food supply chain. This review highlights the expanding uses of DT in livestock farming, underscoring its rapid evolution and transformative potential. Moreover, DT technology enhances livestock management by enabling accurate monitoring of animal health and behavior, predicting diseases, regulating environmental factors such as temperature, humidity, and ammonia levels, and improving productivity and welfare. Additionally, DT supports precision farming, resource optimization, and supply chain management, all of which contribute to greater sustainability and resilience in livestock systems (Arulmozhi et al., 2024).

On the other hand, DT technology plays a crucial role in improving livestock farm operations by enabling the monitoring and prediction of animal behavior, early disease detection, and remote management of farm environments (Hasan et al., 2024). DT acts as the digital and virtual counterpart of a physical entity (Fuller et al., 2020). Moreover, DT technologies are now being utilized across various industries (Errandonea et al., 2020), facilitating the monitoring and prediction of machine conditions and performance (Aivaliotis et al., 2019). In the livestock sector, DT is applied in several areas, such as monitoring animal behavior (Zhang et al., 2023), predicting future behaviors (Han et al., 2022), reducing methane emissions in dairy farms (An and Chen, 2021), optimizing production lines in poultry feed mills (Tarek et al., 2022), managing feeding systems (Raba et al., 2021), and controlling environmental conditions (Jeong et al., 2023).

Tarek et al. (2025) applied DT technology to optimize production operations in a poultry feed mill by developing a digital model that simulates all operational activities. This model improved scheduling efficiency, reduced production time, and optimized energy consumption. The integration of advanced algorithms, such as genetic algorithms and the grey wolf optimizer, enhanced the accuracy of energy consumption calculations, bridging the gap between ideal schedules and actual factory conditions. The benefits of this technology extend beyond efficiency improvements and cost reductions; it also fosters sustainable manufacturing practices. In higher education, DT can be a powerful tool for training poultry science students. By using DT, students can gain hands-on experience in understanding how modern technologies are applied to manage poultry farms and feed mills. They can simulate real-world environments, assess environmental impacts, and analyze real-time data, providing them with valuable practical learning opportunities that enhance their skills and prepare them for careers in the industry (Tarek et al., 2025).

On the other hand, Jia et al. (2025) demonstrated that the use of DT technology in poultry farms can effectively monitor environmental conditions within layer houses without the need for extensive sensor deployment. By utilizing historical data and real-time reference points, advanced computational models were employed to simulate and predict temperature distributions. Field testing revealed that the virtual data closely aligned with actual sensor readings, with an average absolute error of less than 0.25°C. These results underscore the potential of virtual sensing as a reliable and cost-effective solution to support the development of intelligent DT technology in modern poultry production.

In a study conducted by Emmert et al. (2024), DT technology was used to create highly accurate three-dimensional models of the keel bone in laying hens, utilizing quantitative computed tomography scans. These digital replicas allowed researchers to visually monitor and assess the progression of keel bone damage over time in cage-free housing systems. By employing DT, they were able to precisely identify the severity, type, and location of skeletal injuries during different growth and production stages, providing deeper insights into the onset of deviations and fractures. This method offered a non-invasive, objective alternative to traditional assessment techniques, enhancing the understanding of skeletal health dynamics in modern poultry production.

The rapid advancement of digital technologies has opened new avenues for innovation in higher education, particularly in disciplines that require practical and applied expertise, such as poultry production. Among these technologies, DT stands out as a powerful tool capable of transforming traditional educational models into dynamic, data-driven learning environments. By creating live virtual replicas of real poultry production systems, DT enables students to explore complex processes, analyze operational data, and simulate real-world scenarios without the constraints of physical infrastructure or biosecurity risks. This integration not only enhances the learning experience but also bridges the gap between academic knowledge and labor market demands. Pedagogically, DT shift learning from passive theory to active, interactive experiences, allowing students to connect concepts with practical applications, develop critical thinking, and strengthen decision-making and data analysis skills. These competencies align closely with educational outcomes and industry expectations, making DT a valuable tool in preparing students for professional practice in poultry production. The following points outline how DT technology can be strategically employed to support higher education objectives in the context of poultry production:

***Creating a Realistic Virtual Learning Environment.*** DT technology is expected to provide an integrated virtual simulation that accurately mirrors the operational processes of poultry farms and feed mills, including flock movement, ventilation systems, scheduling, and supply chains. This environment serves as an effective learning tool, enabling students to interact with a realistic model without physically being present at production sites. It overcomes geographical and time constraints associated with traditional training, while reducing costs and eliminating risks linked to real-world experimentation.

***Integrating Modern Technologies into Academic Curricula****.* The use of DT technology could have a positive significant impact on the integration of advanced technologies such as IoT, big data analytics, AI, and cloud computing into educational programs. Students not only gain insights into biological processes but also learn how to collect, process, analyze, and interpret real-time data to make informed technical decisions. This integration equips graduates with the digital competencies needed to keep pace with the technological transformation in the poultry production sector.

***Enhancing Decision-Making Skills.*** DT technology is expected to enhance students’ access to real-time or near-real-time data on key production metrics, including feed intake, feed conversion ratios, mortality rates, and environmental conditions within poultry farms. Such exposure allows them to perform predictive analyses and test alternative decisions in a risk-free virtual setting. This hands-on experience enhances their ability to make informed operational decisions and evaluate outcomes in terms of efficiency and productivity, skills essential for future managers and operators.

***Fostering Research and Development.*** DT technology is expected to serve as an ideal platform for conducting research without the need for costly physical infrastructure. Students and faculty researchers can simulate changes in feeding strategies, production scheduling, or the design of ventilation and lighting systems, and evaluate their effects on performance and profitability. These simulations support frequent, precise, and rapidly adjustable experiments, improving research efficiency and reducing the time needed to reach reliable conclusions.

***Supporting Interactive Learning and Continuous Assessment***. DT technology will offer mechanisms for tracking student interaction within the simulation environment and evaluating their decisions against predefined performance indicators, such as growth rate, feed cost, or animal welfare scores. These mechanisms provide real-time, detailed feedback that helps assess student performance objectively and continuously. Academic institutions can use DT-based simulations as practical assessment tools to measure professional competencies, not just theoretical knowledge, thereby enhancing learning quality and outcome reliability.

***Achieving Sustainability Goals and Professional Development***. One of the most valuable aspects of using DT technology is that it will be able to help students evaluate the environmental impact of poultry production activities, such as water and energy consumption and emissions of ammonia and methane. Through data analysis, students develop a deeper understanding of the link between production efficiency and environmental sustainability. This fosters awareness of responsible agricultural practices and prepares students to take on leadership roles in their fields, particularly as the global industry shifts toward more environmentally sustainable food systems.

### The impact of implementing DT-based educational framework on enhancing student engagement, improving learning experience, and increasing job market readiness

Understanding the impact of DT technology across different educational stages is crucial for its successful integration into higher education programs. When properly implemented, DT technology is expected to meet the needs of prospective students, current learners, and recent graduates by providing visual and interactive tools to build foundational knowledge, bridging theory with practical application through realistic simulations, and enhancing advanced skills aligned with industry requirements. Therefore, the use of DT technology is anticipated to have a positive impact as a versatile and scalable tool that effectively supports students at varying levels of experience and readiness.

Moreover, Ezeoguine et al. (2024), reported that DT technology plays a significant role in enhancing collaboration between students and educators, enabling them to engage in shared learning experiences and collaborative projects. This technology provides students with opportunities to interact with innovative educational content, participate in virtual labs, and conduct experiments outside traditional classroom hours. Moreover, it offers hands-on experience in virtual environments, allowing students to explore and interact with complex concepts and phenomena in an interactive manner. In light of these findings, the researchers suggested the creation of partnerships between educational institutions, technology companies, and government agencies. These collaborations would facilitate the sharing of resources, expertise, and funding, thereby effectively supporting the integration of DT technology into the educational process (Ezeoguine et al., 2024).

In the context of poultry production education, modernizing teaching approaches is crucial for influencing graduates' ability to assist poultry companies in overcoming challenges. Additionally, it prepares them to enter the workforce, equipping them to contribute effectively and transparently to company management. Furthermore, graduates can play an active role in community development as consumers gain more insight into their food production process. This educational advancement can empower graduates to become engaged and informed global citizens (Fanatico et al., 2024).

The shortage of graduates pursuing careers in the poultry industry is largely attributed to a lack of awareness and interest. Promoting agricultural literacy could be a key strategy in encouraging greater engagement with future opportunities in poultry science. As such, programs introduced during pre-university education have the potential to significantly enhance agricultural literacy, while also serving as valuable educational resources (Marks et al., 2021).

Erickson et al. (2019) demonstrated that public awareness of the poultry industry remains limited, despite the strong influence of public perception on consumer behavior and the appeal of related academic and career pathways. To address this challenge, an online educational program integrating poultry science with STEM (Science, Technology, Engineering, and Mathematics) disciplines was evaluated to enhance students’ knowledge and interest in the field. The results indicated a marked improvement in students’ understanding of the industry and increased awareness of career opportunities. The evaluation also revealed varying levels of student engagement with the program content, offering valuable insights for the development of more effective educational resources that foster skill development and enhance the overall image of the poultry industry.

Moreover, the online poultry education program has proven beneficial in improving both knowledge and attitudes toward the poultry industry. The results indicated that learner-centered educational programs can create positive learning experiences for many high school students interested in poultry. However, there remains a pressing need for further research to explore how to make poultry science more relevant to students, which would help improve overall agricultural literacy and better meet the workforce demands in the poultry industry (Erickson et al., 2019).

In light of the above, the use of DT technology can be considered one of the factors influencing the increased interest of students in enrolling in poultry science programs. By providing interactive simulations of production processes, this technology helps students explore complex concepts in a virtual environment, enhancing their understanding of the sector and motivating them to make informed academic decisions. This can be explained as follows:

***Increased Interest in the Field****.* DT technology is expected to provide prospective students with immersive, interactive simulations of poultry production processes, offering a hands-on experience of what the industry entails. These virtual models replicate real-world scenarios, allowing students to explore various aspects of poultry farming, from breeding and nutrition to disease management and environmental factors. This realistic exposure increases their understanding of the field and fosters a deeper curiosity about poultry science, encouraging them to pursue a career in the industry. By engaging with the technology, students can better comprehend the complexity and significance of modern poultry production, which enhances their motivation to enroll in related academic programs.

***Positive Early Impressions.*** Early interactions with advanced applications of DT technology, such as smart farm models, virtual monitoring of animal health, and predictive analytics for disease management, are expected to create a strong initial impression of the field. These cutting-edge tools signal that poultry science is not just a traditional agricultural discipline, but a modern, technologically advanced sector that integrates innovation and digital transformation. Exposure to these advanced tools early on reshapes students' perceptions of the field, presenting it as a forward-thinking career path that embraces automation, data-driven decision-making, and sustainable practices. This positive first impression plays a critical role in attracting students who may otherwise be unaware of the dynamic nature of the poultry industry.

***Informed Academic Decisions.*** The ability to engage with realistic digital simulations prior to enrolling in an academic program can have a significant impact by enabling prospective students to more accurately assess their suitability for the field. Through these immersive experiences, students can explore different areas of poultry production in a controlled, virtual environment, gaining clarity about their specific interests and career aspirations. This hands-on exposure helps them make well-informed decisions about whether they want to pursue poultry science as a major or explore other career paths. By engaging with DT models, students can align their academic choices with their personal interests and strengths, leading to more confident, purposeful decisions about their future education.

On the other hand, With the increasing demand for higher education to meet job market needs, technologies like DT have emerged as innovative solutions to enhance learning quality. The DT-based educational framework offers interactive, real-world simulations that deepen students’ understanding of production processes and boost practical skills. Additionally, it cultivates critical thinking, problem-solving, and data-driven decision-making, effectively preparing students for careers in poultry production. Moreover, DT technology holds great potential to enhance learning by enabling educators to design immersive, context-rich environments and collect detailed learner data for more accurate assessments. As a simulation-based tool, DT supports diverse educational applications, fostering deeper engagement. However, ongoing research is essential, and educators are encouraged to conduct experimental and design-based studies to unlock further innovations and advance teaching and learning (Selim et al., 2024).

Moreover, DT technology is revolutionizing educational technology by improving learning outcomes, boosting motivation and engagement, developing practical skills, offering data-driven insights, and supporting remote and hybrid learning. As DT advances, its use in education is expected to grow, enabling innovative, interactive, and personalized teaching methods. In classrooms, DT facilitates safe experimentation, real-time feedback, collaborative learning, and extends education beyond traditional settings. However, challenges such as high costs, technological complexity, data privacy, resistance to change, training needs, digital divides, and scalability must be addressed. Successful integration of DT requires close collaboration among educators, technologists, policymakers, and the education community to overcome these obstacles (Ezeoguine et al., 2024).

In the domain of poultry science education, the need to prepare students for addressing socio-environmental issues such as food insecurity, climate change, and biodiversity loss makes DT integration even more vital. Sustainable poultry production can help address many critical issues, and educating undergraduate students in this field is essential for developing a responsible and skilled workforce. These students become engaged citizens who contribute to community-based food systems, help companies address sustainability challenges, and provide a global perspective on sustainable development. Therefore, poultry science curricula must reflect both environmental and societal priorities. In the US, such curricula are commonly housed within animal science departments at land-grant universities, with some institutions maintaining dedicated poultry science departments. Career readiness is a central pillar of these programs, ensuring graduates are equipped to succeed in the poultry industry (Fanatico et al., 2024).

The integration of the DT concept in universities serves multiple educational goals: virtualizing academic environments, enhancing learning experiences, strengthening technical training, preparing students for the job market, gamifying educational content, and reducing costs associated with labs and equipment. One widely adopted application is the virtual representation of expensive technical lab environments. A case study by Zacher (2020) found that using DT in a Master's Applied Sciences program led to a 26 % reduction in lab costs. Additionally, the interdisciplinary nature of DT development supports project management skill building. Hagedorn et al. (2023) demonstrated this application. Additionally, DT also helps assess student effort and motivation in engineering courses. For example, students in a Control Systems Simulation course developed DT of a Programmable Logic Controller, which enhanced their engagement and responsibility toward learning (Liljaniemi and Paavilainen, 2020).

In robotics education, DT enables the virtualization of training centers to overcome lab space limitations. Erdei et al. (2022) replicated a Robotic Industrial Arm lab using DT and compared two student groups, one using DT and the other relying on documentation. Students trained with DT outperformed their peers, highlighting the effectiveness of the technology in boosting learning outcomes (Bachmann et al., 2024).

Research also supports the use of DT in creating virtual laboratories that simulate real lab environments, enabling students to perform experiments, make observations, and analyze data digitally. These simulations are especially useful where physical access is limited. DT enable modeling of complex scientific phenomena across multiple fields like engineering, biology, and chemistry (Dietz et al., 2021; Alsaleh et al., 2022; Guc et al., 2021).

For educators, DT simulates classroom scenarios, allowing instructors to refine teaching methods and student interaction strategies. This form of training improves pedagogical skills and teaching quality (Siyan et al., 2021). Additionally, DT supports research by providing digital representations of study subjects or concepts, enabling efficient data collection and simulations without relying on physical resources (Vogel-Heuser et al., 2021).

By integrating DT technology into multiple educational domains, institutions can enhance learning experiences, offer adaptive services, and improve student satisfaction with academic and administrative support (Chande, 2024). Students also contribute to institutional reputation through feedback and advocacy, influencing institutional competitiveness (Williams et al., 2017; Chen, 2016; Tung et al., 2017).

On the other hand, DT technology is expected to enhance the learning experience of poultry science students by providing realistic and secure simulations that enable direct interaction with production environments. This technology will empower students to practically apply theoretical concepts, analyze data, and make decisions based on real information, as well as study the impact of factors such as nutrition, environment, and health on productivity. Consequently, the technology will improve students’ professional readiness and elevate their technical skills related to modern technologies in the poultry industry as follows:

***Enhancing Understanding of Theoretical Concepts****.* Virtual models of real poultry environments are expected to provide students with a powerful platform to understand and analyze the effects of various factors, such as nutrition, environment, and health, on productivity. These models allow students to visualize complex processes that may be difficult to explain in traditional classrooms, enhancing their ability to link theory with practical application in a live production setting. Additionally, students can analyze results in real-time, helping them gain a deeper understanding of how management decisions affect overall performance.

***Increasing Engagement and Motivation***. The interactive learning environments provided by DT technology is expected to enable students to directly engage with production scenarios, motivating them to actively participate in the learning process. Students face challenges in an interactive simulation environment that requires them to think critically and make data-driven decisions. This type of learning boosts students' personal motivation and encourages innovation and future thinking, making them more prepared to adapt to the rapid changes occurring in the poultry industry.

***Developing Technical Skills***. By working with live data and digital interfaces, students develop skills in areas such as data analysis, programming, and IoT technologies. These skills are essential in today’s technologically evolving world. Interacting with digital systems enables students to apply theoretical knowledge practically, while also strengthening their ability to integrate technology with agricultural production. Students who master these tools are better equipped to use technology to develop innovative solutions in the poultry sector.

***Better Preparation for the Workforce****.* DT technology may be effective in providing students with a unique opportunity to gain hands-on experience using tools and scenarios relevant to the poultry industry, thereby enhancing their readiness for the demands of the job market. Students working with this technology acquire both technical and practical skills, enabling them to address real-world challenges they may encounter in the workplace, such as optimizing production, effectively managing resources, and solving problems using data. This better prepares them to work in digital and advanced environments.

On the other hand, the results of applying this technology in poultry science education for the recent graduates can be summarized as follows:

***Improved Career Readiness.*** Graduates trained with DT systems are expected to be better prepared to work in advanced digital environments, such as smart farms and agricultural technology companies. These graduates possess a deep understanding of the technologies shaping the future of the poultry industry, making them ready to operate in environments that require dealing with advanced tools utilizing artificial intelligence, data analytics, and automation.

***Increased Employability****.* Hands-on experience with digital technologies is expected to give graduates a competitive edge in the job market, particularly in fields that require skills in predictive analytics, IoT, and automated systems. Graduates who possess these skills become more attractive to companies seeking employees capable of using technology to enhance productivity and make informed decisions. This provides them with greater opportunities for specialized roles in data analytics and modern agricultural technologies.

***Potential to Drive Industry Innovation***. Equipped with the technological insight gained from training with DT, graduates may significantly contribute to the development and improvement of the workplaces they join. They can offer innovative solutions in their work environments or even lead their own projects in tech-integrated agribusiness ventures. Graduates trained in these technologies are capable of forecasting future trends and contributing to the industrial shifts that are redefining the poultry industry with digital solutions and modern technologies.

***The Key Challenges and Opportunities in Integrating DT Technology into Poultry Education and Livestock Production Systems.***

The integration of DT technology into agricultural systems, particularly in poultry education and livestock production, represents a transformative step towards digitization and sector modernization. This advanced technology enables simulation, monitoring, and real-time analysis of complex biological and operational systems, offering vast potential to enhance productivity, efficiency, and educational engagement. However, despite the promising benefits of this technology, its adoption in these contexts remains limited due to a range of technical, financial, infrastructural, and educational challenges. Understanding these barriers and exploring practical, interdisciplinary solutions is essential to developing flexible and future-ready educational frameworks that can prepare students to navigate the transformations in the field of digital agriculture.

Despite the transformative potential of DT technology, its adoption in animal production, including poultry production, continues to face numerous challenges. These include the absence of standardized frameworks and regulatory guidelines, a shortage of skilled professionals, underdeveloped software infrastructure, and serious concerns regarding data security and ownership. These challenges are also reflected in educational environments, where the lack of operational models and practical case studies hinders the integration of DT technologies into poultry science curricula. Therefore, there is an urgent need for empirical evidence, supportive policy measures, and practical educational frameworks to promote broader adoption and effective training (Arulmozhi et al., 2024).

In addition to the above, several other obstacles continue to hinder the widespread deployment of DT technology. These include high implementation costs, insufficient technological expertise, concerns about data privacy, weak models for human-animal interaction, and sustainability issues. Addressing these challenges in an educational context requires a multidisciplinary approach and broad collaboration. Moving forward, research efforts should focus on developing robust protocols for data collection and integration, enhancing AI models, analyzing cost-benefit ratios, monitoring animal behavior and welfare, assessing environmental impacts, ensuring cybersecurity, strengthening stakeholder training that includes educators and students, and expanding the scope of DT applications. These efforts are expected to improve the accuracy of DT models, support well-informed investment decisions, ensure animal welfare, optimize resource management for sustainable farming, increase stakeholder trust, raise DT adoption rates, and ultimately enhance livestock productivity. Governments and the private sector must provide adequate funding and support collaborative research initiatives, as these are essential for expanding the use of DT technology in livestock farming systems and for developing advanced educational programs that prepare students for the era of digital agriculture (Hasan et al., 2024).

## Conclusion

The integration of DT technology in poultry education is expected to has a significant impact on various aspects of the educational process, extending its influence from students to educators and scientific research. For incoming students, the use of this technology is anticipated to provide an interactive learning environment that allows them to engage with simulations of production processes and practical components in laboratories, enhancing their understanding of both theoretical and practical aspects in an integrated manner.

Additionally, it's expected to motivate them to make more informed and confident academic decisions. For current students and recent graduates, DT technology is projected to help bridge the gap between theoretical knowledge and practical application by offering simulated, real-world environments. Graduates will also be able to leverage this technology to refine their professional skills and stay updated on the latest developments in the poultry industry. Furthermore, the impact of DT technology is expected to extend to educators by empowering them to deliver more interactive and engaging educational content, thereby improving the learning experience and aligning it more closely with real-world industry scenarios. It will also offer educators opportunities to enhance their teaching methodologies through digital applications, opening new pathways for professional development.

In the realm of scientific research, DT technology is poised to provide tremendous opportunities for studying phenomena and analyzing data within realistic experimental environments. Poultry researchers can utilize DT technology to simulate and analyze data related to poultry production, behavior, and the interactions between the environment and agricultural practices. Moreover, this technology is anticipated to enable simulations and predictive analyses without the need for physical experiments, thus saving time and resources while fostering innovation, expanding research scope, and improving cost efficiency associated with laboratories and research facilities.

Despite these numerous benefits, the adoption of DT technology in poultry science education and research faces challenges such as a lack of technological infrastructure, high costs, and the need for specialized training for educators. Therefore, collaboration between educational institutions, governments, and private companies is essential to provide the necessary financial and technical support, along with offering training programs for educators and researchers. This collaboration will contribute to the effective integration of DT technology into educational curricula and research programs, ultimately advancing the field and preparing students for a promising future in poultry science.

The current study recommends that higher education institutions systematically adopt DT technology within poultry science programs by developing curricula that incorporate interactive virtual simulations as a core educational tool. This approach aims to enhance students’ practical and cognitive understanding, encourage enrollment in poultry programs, and improve graduates’ readiness for the job market. Additionally, the study emphasizes the importance of providing technical and training support for faculty members and fostering collaboration with specialized smart agriculture technology providers to ensure the effective and sustainable integration of this technology into the educational process.

## Funding

This work was supported by the Deanship of Scientific Research, Vice Presidency for Graduate Studies and Scientific Research, King Faisal University, Saudi Arabia (Grant No. KFU251742).

## Disclosures

All authors declare that there are no conflicts of interest regarding the publication of this paper.
